# Multiple huge “cluster” and “galaxy” signs on chest radiography in a patient with pulmonary tuberculosis

**DOI:** 10.1002/rcr2.398

**Published:** 2019-01-25

**Authors:** Miku Oda, Takeshi Saraya, Tatsuya Shirai, Narishige Ishikawa, Masachika Fujiwara, Hajime Takizawa

**Affiliations:** ^1^ Department of Respiratory Medicine Kyorin University School of Medicine Mitaka Japan; ^2^ Department of Pathology Kyorin University School of Medicine Mitaka Japan

**Keywords:** Chest radiography, cluster sign, computed tomography, galaxy sign, pulmonary tuberculosis

## Abstract

A 62‐year‐old healthy man presented to our hospital due to a persistent fever of up to 38°C for one week. Thoracic computed tomography showed right pleural effusion with multiple large nodules up to 7 cm in diameter composed of numerous discrete small nodules like fireworks, the so‐called “cluster” signs. Some of the large nodules had a hyper‐dense portion centrally surrounded by partially discrete small nodules, not as densely assembled, suggestive of the “galaxy” sign. The repeated acid‐fast sputum smears and both bronchial washings were all negative for *Mycobacterium tuberculosis*, but the acid‐fast culture of sputum taken soon after the first bronchoscopy, and pleural fluid, turned out to be positive for *M. tuberculosis* at six weeks after admission. The present case clearly demonstrates that the “galaxy” and “cluster” signs are red herring signs of the low rates of isolating *M. tuberculosis*, which should be differentiated from pulmonary sarcoidosis.

## Introduction

Two distribution patterns of small pulmonary nodules on thoracic computed tomography (CT) have been recognized as the “galaxy” and “cluster” signs, and are characteristic findings in pulmonary tuberculosis (TB) and sarcoidosis. The “galaxy” sign was first reported in 2002 by Nakatsu et al. 1 in patients with pulmonary sarcoidosis, and is defined as (1) nodules with irregular margins consisting of numerous small nodules with relatively distinct margins, and (2) low attenuation area within the nodules. In 2009, Ortega et al. 2 described the “sarcoid cluster sign,” an accumulation of distinct small nodules. However, both “galaxy” and “cluster” signs can be seen in pulmonary TB as well 3, 4. In this article, we present a unique case of active pulmonary TB, with multiple huge “cluster” signs like fireworks, together with multiple “galaxy” signs.

## Case Report

**Figure 1:**
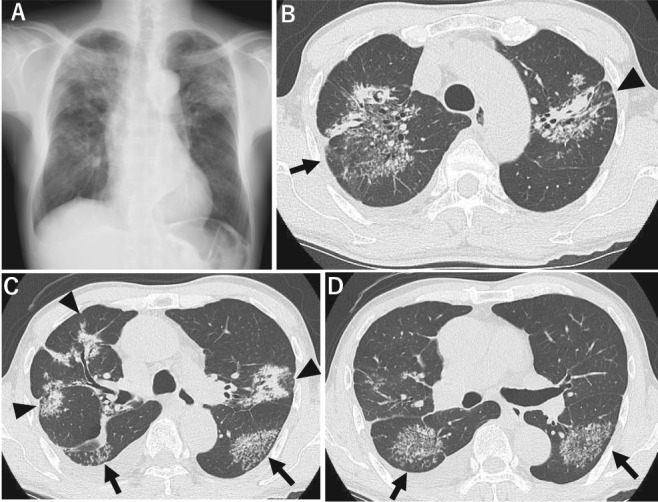
Thoracic radiography showed infiltrates in the entire right lung field and left upper lung field (A). Thoracic computed tomography demonstrated the multiple “cluster” signs, consisting of an accumulation of abundant discrete small nodules like fireworks (B–D, arrows). Multiple “galaxy” signs were also noted in (B) and (C) (arrow heads), which had dense central portions surrounded by partially discrete small nodules (B and C, arrow heads).

A 62‐year‐old man visited our hospital due to a persistent fever of up to 38°C for one week. He had no significant medical history. He worked as a teacher at the university, and was an ex‐smoker with a 14 pack‐year history. He appeared to be well, and his vital signs were normal except for a low‐grade fever of 37.5°C. However, chest radiography showed infiltrates in the upper lung fields bilaterally, and in the right middle to lower lung fields (Fig. 1A). The infiltrates were confirmed on the thoracic CT, which showed multiple large nodules up to 7 cm in diameter (Fig. 1B, arrow), which were in turn composed of numerous discrete small nodules like fireworks (Fig. 1B–D, arrows), the so‐called “cluster” signs. Some of the large nodules had a hyper‐dense portion centrally as a result of the coalescence of smaller nodules surrounded by partially discrete small nodules, not as densely assembled, suggestive of the “galaxy” sign (Fig. 1B, C, arrow heads). Thoracic CT showed no apparent mediastinal lymphadenopathy.

Based on a suspicion of TB or sarcoidosis, three samples of sputum or gastric contents were obtained on three separate days, tested using smears and cultures, and the results were negative for acid‐fast bacilli in the outpatient setting. Additionally, the first bronchial brushings obtained from the right upper lobe (S1) and left upper lobe (S1 + 2) showed no evidence of *Mycobacterium tuberculosis* on cytology, polymerase chain reaction, and acid‐fast culture. Therefore, 10 days after his first visit to our hospital, he was admitted to the respiratory department.

On the day of admission (Day 1), right thoracentesis was performed, which showed elevated lactate dehydrogenase levels of 1254 IU/L, total protein level of 5.1 g/dL, and glucose levels of 100 mg/dL, consistent with an exudative pleural effusion. The pleural fluid showed an increased total cell count of 1250 cells/μL, with a lymphocytic predominance (neutrophils 15%, lymphocytes 73%, monocytes 11%, and other cells 1%). Furthermore, marked elevation of adenosine deaminase levels in the pleural fluid (108 U/L) was noted. Serum laboratory test for TB, T‐SPOT, was positive. Those results lead to the tentative diagnosis of bilateral pulmonary TB pleuritis on the right side, and anti‐TB medication was commenced on Day 8.

**Figure 2:**
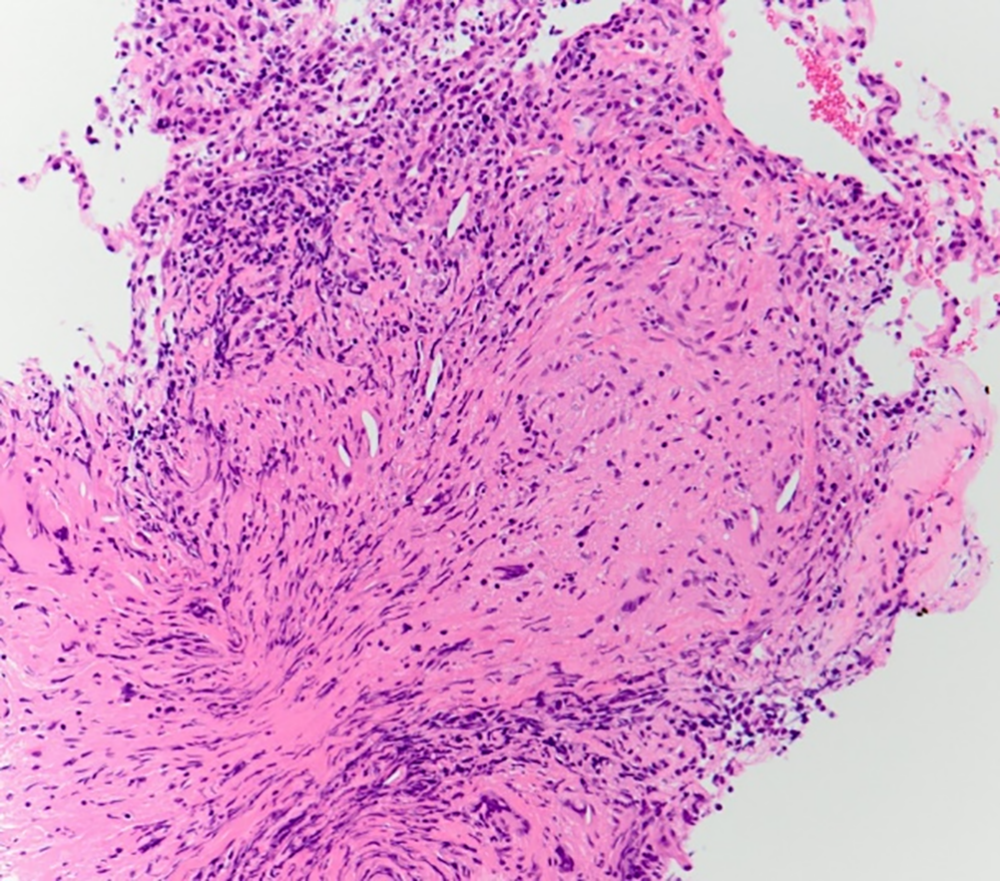
On haematoxylin‐eosin stain (400×), transbronchial biopsy specimens showed non‐caseating granulomas.

The second bronchoscopy was performed on Day 4. The bronchoalveolar lavage (BAL) fluid obtained from the right middle lobe (B4) demonstrated an elevated total cell count (5.5 × 10^5^ cells/mL), with lymphocytic predominance (lymphocytes 41%, macrophages 58%, and eosinophils 1%). The CD4‐to‐CD8 ratio of the lymphocytes in the BAL fluid was elevated at 5.87. Additionally, acid‐fast culture of the BAL fluid was negative. Furthermore, on haematoxylin and eosin staining, the transbronchial biopsy specimens obtained from the right upper lobe (B3) and middle lobe (B4) showed non‐caseating granulomas (Fig. 2). Taken together, these results favour the diagnosis of pulmonary sarcoidosis, rather than TB.

However, at Day 21, acid‐fast culture from the pleural effusion sample proved to be positive for *M. tuberculosis.* At Day 42, acid‐fast culture of sputum taken soon after the first bronchoscopy was positive for *M. tuberculosis*, but was negative in all materials derived from both bronchoscopic procedures.

## Discussion

The presence of the “cluster” sign and the “galaxy” sign on thoracic CT should suggest the possibility of pulmonary sarcoidosis, TB, or silicosis. This case had no occupational history increasing his risk for silicosis; thus the most likely diagnosis was sarcoidosis or TB.

Our previous study showed that the “galaxy” sign was considerably more common in patients with pulmonary sarcoidosis than in patients with pulmonary TB, and was associated with a younger age and low BAL fluid CD4/CD8 ratio, but was not associated with disease severity in sarcoidosis 3. Furthermore, the median number of the “galaxy” sign was three per patient, with a statistically significant distribution more commonly involving the upper lung fields than the lower lung fields, as in the present case. In contrast, the number of “galaxy” signs in cases with active TB is usually a single cluster in the superior segment of the lower lobe (as shown in Fig. 1D), without lymphadenopathy or tree‐in‐bud patterns 4. From this perspective, the high CD4/CD8 ratio of the lymphocytes in the BAL fluid, together with multiple “galaxy” signs or “cluster” signs, without definite isolation of *M. tuberculosis* in the respiratory samples, suggests the diagnosis of pulmonary sarcoidosis. However, the presence of the “cluster” signs in the superior segment of the lower lobes (Fig. 1C, D, arrows) suggests the diagnosis of pulmonary TB 4. Japan is a medium‐burden pulmonary TB country; therefore, interpretation for positive result of T‐SPOT was equivocal in this case.

Thus the complex radiological features and the clinical findings include the BAL fluid in this case cannot confirm the final diagnosis, despite consensus from physicians and radiologists, without microbiological analysis.

The “cluster” sign corresponded to non‐caseating, non‐coalescing granulomas located in the lymph vessels, with a predominance of CD4+ lymphocytes, and no fibrosis 2. It is important to note that the “cluster” sign does not always indicate sarcoidosis, as it can be seen in TB as well 4, as shown in our case (Fig. 1B–D, arrows). This suggests that it is difficult to interpret the modes of spread of small nodules using high‐resolution CT, such as perilymphatic (common in sarcoidosis) or transbronchial (typically recognized as the tree‐in‐bud sign, suggestive of TB). In terms of correlation between pathology and radiological signs, the “galaxy” sign usually has an aggregation of numerous coalescent small nodules centrally presented as a large nodule, whereas the peripheral area usually has partially coalescent small nodules corresponding to peripheral low‐attenuation spots on CT 1, 5, as seen in our case (Fig. 1B, C, arrow heads). In contrast, “cluster” signs consist of a large number of discrete small nodules without coalescence, which contain caseating or non‐caseating granulomas.

On microbiological assessment, some TB experts have stated that the rate of positive acid‐fast cultures and smears, obtained from sputum and/or bronchial washing, is extremely low in cases in which the “galaxy” sign or “cluster” sign are seen. This raises the hypothesis that the “galaxy” sign and “cluster” signs themselves were “red herrings” when the physicians considered the possibility of pulmonary TB, because each small nodule in these signs corresponded to the well‐established granuloma, which were less likely to isolate *M. tuberculosis.* In other words, both the “galaxy” and “cluster” signs on high resolution CT might be considered as “red herring” signs of the low possibility of isolating *M. tuberculosis.* Indeed, the present case showed that the repeated acid‐fast sputum smears and both bronchial washings were all negative for *M. tuberculosis*, but only the acid‐fast culture of sputum taken soon after the first bronchoscopy, and pleural fluid, turned out to be positive for *M. tuberculosis* at six weeks after admission.

Thus, the “galaxy” sign and “cluster” sign are unique and infrequent signs, useful in narrowing the differential diagnosis; however, clinicians should be aware of the low rates of *M. tuberculosis* isolation.

### Disclosure Statement

Appropriate written informed consent was obtained for publication of this case report and accompanying images.
